# Supplementary material for: Exploring Gender Differences in the Effects of Diet and Physical Activity on Metabolic Parameters
Source: Nutrients. 2025 Jan 20;17(2):354. doi: 10.3390/nu17020354 (PMC11768364; doi:10.3390/nu17020354)
Supplement: Supplementary file 1 [file nutrients-17-00354-s001.zip › nutrients-3428437-supplementary.pdf]

Table S1 - Classification of Sports Based on Aerobic, Anaerobic, and Combined Aerobic-Anaerobic Training

Classification	Included Sports
**Aerobic (Aer)**	Acquagym, Bike, Cycling, Hydrobike, Running, Spinning, Swimming, Tapis Roulant, Walking
**Anaerobic (Ana)**	Bodybuilding, Crossfit, Fitness, Golf, Home Workout, Martial Arts, Pilates, Total Body, Wing Chun
**Combined Aerobic and Anaerobic Training (Aer/Ana)**	Beach Volley, Boxe, Dance, Functional Bodybuilding, Functional Training, Soccer, Tennis

Table S1. This table categorizes the sports engaged by participants into three classifications: Aerobic, Anaerobic, and Combined Aerobic and Anaerobic Training. The aerobic classification includes sports focused on endurance and cardiovascular activity, while the anaerobic classification consists of activities primarily involving resistance or high-intensity training. The combined classification refers to sports incorporating both aerobic and anaerobic components in training routines.

Table S2. - Descriptive Statistics, Deltas, and p-values for Metabolic Parameters at Baseline and After 6 Months (T6), Stratified by Gender.

	**Total Mean**	**SD**	**Males Mean**	**SD**	**Females Mean**	**SD**	**Δ total**	**Δ total SD**	**Δ males**	**Δ males SD**	**Δ females**	**Δ females SD**	**p Δ total**	**p Δ male**	**p Δ females**	**p Δ males vs females**
Glucose (mg/dL)	96.3	14.1	98.4	16.3	94.1	10.8										
Glucose 6 months (mg/dL)	91.5	12.0	93.3	12.9	89.5	10.7	-4.8	10.2	-5.1	10.7	-4.6	9.6	**<0.0001**	**<0.0001**	**<0.0001**	0.6219
Total cholesterol (mg/dL)	204.4	42.3	204.0	44.7	204.8	39.6										
Total cholesterol 6 months (mg/dL)	184.3	36.3	179.7	35.3	189.5	37.0	-20.1	31.8	-24.3	32.3	-15.3	30.6	**<0.0001**	**<0.0001**	**<0.0001**	**0.0481**
HDL cholesterol (mg/dL)	54.8	15.1	48.3	11.6	62.5	15.3										
HDL cholesterol 6	53.7	14.7	47.8	12.3	60.5	14.4	-1.1	9.9	-0.5	9.8	-2	10.1	**0.0268**	**0.3455**	**0.0273**	0.2882

months (mg/dL)																
LDL cholesterol (mg/dL)	130.1	41.3	132.3	42.8	127.3	39.5										
LDL cholesterol 6 months (mg/dL)	112.4	33.5	112.3	34.9	112.6	31.8	-18.2	21.1	-20	25.7	-14.7	30.8	**0.0015**	**<0.0001**	**0,0011**	**0.0379**
Triglycerides (mg/dL)	115.0	71.9	132.5	83.4	95.4	49.8										
Triglycerides 6 months (mg/dL)	100.9	45.8	108.3	47.6	92.2	42.2	-14.1	58.9	-24.2	52.7	-3.2	43	**<0.0001**	**<0.0001**	**0.0018**	0.2283
AST (U/L)	25.3	13.2	28.9	15.5	21.3	8.3										
AST 6 months (U/L)	21.7	7.5	23.9	8.1	19.1	5.6	-3.6	13.1	-5	15.9	-2.2	8.5	**0.0003**	**0.0049**	**0.0133**	0.2164
ALT (U/L)	32.7	26.7	39.0	28.4	25.6	22.7										
ALT 6 months (U/L)	24.4	13.1	27.9	15.3	20.4	8.4	-8.2	29.8	-11.1	28.1	-5.2	24.3	**<0.0001**	**<0.0001**	**0.044**	0.2289
Creatinine (mg/dL)	0.92	0.21	1.02	0.20	0.8	0.15										
Creatinine 6 months (mg/dL)	0.89	0.20	0.98	0.17	0.77	0.16	-0.03	0.16	-0.04	0.19	-0.03	0.12	**0.0312**	0.1378	0.0842	0.9083

Table S2. Descriptive statistics, deltas (Δ), and p-values for metabolic parameters at baseline (T0) and after six months (T6), stratified by gender. Parameters include fasting glucose (mg/dL), total cholesterol (mg/dL), HDL cholesterol (mg/dL), LDL cholesterol (mg/dL), triglycerides (mg/dL), AST (U/L), ALT (U/L), and creatinine (mg/dL). Mean and standard deviation (SD) values are reported for the overall group, males, and females at T0 and T6. Δ indicates changes from T0 to T6, with mean ± SD values calculated for the overall group, males, and females. p-values are provided for comparisons of Δ within groups (overall, male, and female) and between genders.
